# Profiling of phytochemical and antioxidant activity of wild mushrooms: Evidence from the in vitro study and phytoconstituent's binding affinity to the human erythrocyte catalase and human glutathione reductase

**DOI:** 10.1002/fsn3.2650

**Published:** 2021-11-02

**Authors:** S. M. Moazzem Hossen, Mohammad Shahadat Hossain, A. T. M. Yusuf, Priya Chaudhary, Nazim Uddin Emon, Pracheta Janmeda

**Affiliations:** ^1^ Department of Pharmacy Faculty of Biological Science University of Chittagong Chittagong Bangladesh; ^2^ Department of Pharmacy University of Science and Technology Chittagong Bangladesh; ^3^ Department of Bioscience & Biotechnology Banasthali Vidyapith Banasthali India; ^4^ Department of Pharmacy Faculty of Science and Engineering International Islamic University Chittagong Chittagong Bangladesh

**Keywords:** antioxidants, catalase, DPPH, glutathione reductase, in silico, mushrooms

## Abstract

This study was undertaken to evaluate the appearance of phytochemicals and antioxidant activity of seven wild mushrooms of the University of Chittagong campus. Phytochemical screening was performed using standard methods, whereas DPPH radical scavenging assay was used to elucidate the antioxidant effect. Besides, in silico studies were implemented using the targets of human erythrocyte catalase 3‐amino‐1,2,4‐triazole, human glutathione reductase, and selected compounds. Again, the absorption, distribution, metabolism, elimination and toxicity (ADME/T) analysis has been determined by using online tools. Both *Ganoderma lucidum* (Curtis) Karst. and *Ganoderma applanatum* (Pers.) Pat. showed a significant (*p* < .001) increase in the percentage of scavenging activity at 400 μg/ml concentration when compared with ascorbic acid. The methanol extract of *G*. *lucidum*, *G*. *applanatum*, and *Rhodofomes cajanderi* (P. Karst.) B. K. Cui, M. L. Han & Y. C. Dai showed strong antioxidant activity with an IC_50_ value. In addition, molecular docking studies of the previously isolated compounds from three selective mushrooms revealed that the targeted compounds along with positive controls were able to interact strongly (range: −3.498 to −8.655) with the enzymes. The study concludes that the *G* *. lucidum*, *G*. *applanatum*, and *R*. *cajanderi* mushrooms can be a strong source in the management of oxidative stress‐induced diseases.

## Graphical abstract

This article aggregates consolidates and validates free radical scavenging activity of seven wild mushrooms along with their phytoconstituent’s binding affinity to the human erythrocyte catalase.

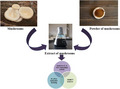

## 1 INTRODUCTION

Oxidation is a vital process in humans that enables the transformation of nutrients such as carbohydrates, protein, and fat into energy (Sánchez, 2016). During this normal metabolic process, reactive oxygen species (ROS) are generated as a by‐product. Highly reactive, unstable, and partially reduced oxygen derivatives such as superoxide radicals (O_2_•—), hydroxyl radicals (•OH), hydrogen peroxide (H_2_O_2_), and singlet oxygen (^1^O_2_) are known as ROS (Chio & Tuveson, 2017). At low‐level, ROS are crucial for various physiological processes and act as secondary messengers (Rajendran et al., 2014). ROS at high concentrations may exert harmful effects on cellular components such as DNA mutations, lipids peroxidation of membrane lipids, and membrane protein damage (Karim et al., 2020). The variation between the creation of ROS and the antioxidant defense capacity of the body is known as *oxidative stress* (Liguori et al., 2018). Oxidative stress is responsible for causing several diseases such as cancer, atherosclerosis, cardiovascular disease, diabetes, and metabolic disorders (Pizzino et al., 2017). Oxidative stress is regarded as an important characteristic for the pathogenesis and development of type 2 diabetes mellitus, but whether it is a simple combination of inflammatory responses or of a clinical entity that is based on different physiological variables is still disputed (Rehman & Akash, 2017). Antioxidants donate electrons to stabilize ROS to prevent cell and tissue damage (Khatua et al., 2013). Catalase is a vital enzyme of the antioxidant system of the human body. It helps to keep the redox stability of the immune system by reducing hydrogen peroxide (Wang et al., 2013). Another essential antioxidant enzyme is glutathione reductase (GR). GR in reduced form controls the ROS at the cellular level (Carlberg & Mannervik, 1985). Endogenous antioxidants and exogenous antioxidants are two known types of antioxidants. The human body makes endogenous antioxidants, which play an important role at low concentrations by scavenging the free radicals to keep maximum cellular functions. However, in case of oxidative stress, these endogenous antioxidants are found to be insufficient to protect the body from the harmful effect of ROS. Diet or dietary supplements may be required as exogenous antioxidants to maintain optimal cellular function (Kurutas, 2016). Nowadays, the industry that is responsible for producing food uses several synthetic antioxidants that have shown carcinogenicity. As a result, there is an urgency to search for antioxidants from natural sources (Abdullah et al., 2012). Recently, consumption of edible mushrooms has increased greatly because they are high in carbohydrates, protein, fiber, essential amino acids, and vitamins while low in fat, cholesterol, sodium, and calories (Rashidi & Yang, 2016). In many cultures, edible mushrooms have been used traditionally as a source of home remedy from long ago due to the presence of biologically active compounds to protect the body from various oxidative stress‐induced diseases (Chen et al., 2012). Several scientific reports have reported the medicinal properties of mushrooms including free radical scavenging, antioxidant (Sánchez, 2017), immunomodulating (Shamtsyan et al., 2004), antitumor (Singdevsachan et al., 2016), antidiabetic (Stojkovic et al., 2019), antihypercholesterolemia (Wasser, 2017), antibacterial, and antiviral effects (Roncero‐Ramos et al., 2017). Hence, the aim of this study was the evaluation of phytochemicals and the antioxidant potential of the wild mushrooms found on the campus of the University of Chittagong.

## 2 MATERIALS AND METHODS

### 2.1 Reagents

1,1‐Diphenyl‐2‐picrylhydrazyl (DPPH) was obtained from Sigma Chemical Co., USA. Ascorbic acid was purchased from *SD* Fine Chemicals Ltd., Biosar, India. Other chemicals of analytical grade were supplied by the Department of Pharmacy, University of Chittagong.

### 2.2 Collection and identification of the mushroom

Seven naturally growing mushrooms including *Lentinus squarrosulus* Mont., *Daldinia concentrica* (Bolton) Ces. & De Not., *Cubamyces lactineus* (Berk.), *Rhodofomes cajanderi* (P. Karst.) B. K. Cui, M. L. Han & Y. C., *Daedaleopsis confragosa* (Bolton) J. Schröt., *Ganoderma applanatum* (Pers.) Pat., and *Ganoderma lucidum* (Curtis) Karst. were collected from different areas of the University of Chittagong campus. Specimens of the mushrooms were identified by Md. Owahidul Alom, Assistant Horticulture Officer, Botanical Garden, Department of Botany, University of Chittagong. The specimen numbers of the mushroom have been given in the Table 1 and preserved in the Department of Pharmacy, University of Chittagong.

**TABLE 1:** Identified mushrooms with the accession number

Mushroom	Family	Accession number
*Ganoderma applanatum* (Pers.) Pat.	Ganodermataceae	2018/004/Fungi/CU/DP
*Ganoderma lucidum* (Curtis) Karst.	Ganodermataceae	2018/005/Fungi/CU/DP
*Lentinus squarrosulus* Mont.	Polyporaceae	2018/007/Fungi/CU/DP
*Daldinia concentrica* (Bolton) Ces. & De Not.	Hypoxylaceae	2018/008/Fungi/CU/DP
*Cubamyces lactineus* (Berk.)	Polyporaceae	2018/009/Fungi/CU/DP
*Rhodofomes cajanderi* (P. Karst.) B. K. Cui, M. L. Han & Y. C.	Fomitopsidaceae	2018/010/Fungi/CU/DP
*Daedaleopsis confragosa* (Bolton) J. Schröt.	Polyporaceae	2018/011/Fungi/CU/DP

### 2.3 Preparation of extract

After shade drying, the mushrooms were milled for efficient extraction. Exactly 100 g of milled mushroom powder was soaked in 500 ml methanol in a clean, sterilized, and flat‐bottomed glass container for 7 days accompanying occasional stirring and agitation at room temperature. It was then filtered using filter papers (Whatman size no. 1). The filtrate was allowed to evaporate the solvent by using a rotary evaporator. These extracts were kept in tightly closed glass containers and stored in the refrigerator for further use. The extracts of different mushrooms were named as follows: MELS, methanolic extract of *L. squarrosulus*; MEDC^1^, methanolic extract of *D. concentrica*; MECL, methanolic extract of *C. lactinea*; MEFC, methanolic extract of *Fomitopsis* *cajanderi*; MEDC^2^, methanolic extract of *D. confragosa*; MEGL, methanolic extract of *G. lucidum*; and MEGA, methanolic extract of *G. applanatum*.

### 2.4 Phytochemical screening

All of the methanol extracts of different mushrooms were qualitatively analyzed for the presence of different chemical groups, such as alkaloids, glycosides, steroids, carbohydrates, tannins, flavonoids, and saponins (Emon, Alam, Uddin Sawon, et al., 2021; Ghosh et al., 2015; Sarker et al., 2016).

### 2.5 Antioxidant activity

Antioxidant activity of different mushroom extracts was carried out using the method of Alam et al. (Alam et al., 2020; S. Alam, Rashid, et al., 2021). Two milliliters of each mushroom extract with different concentrations (12.5, 25, 50, 100, 200, and 400 μg/ml) was mixed with 3 ml of a 0.004% w/v methanol solution of DPPH. Then, the tubes containing the mixture were kept at room temperature for 30 min in a dark place to complete the reaction. The absorbance was taken at 517 nm against using an ultraviolet–visible spectrophotometer (Halo SB‐10 single‐beam spectrophotometer, Dynamica Scientific Ltd., UK). Ascorbic acid was used as a positive control. The capability to scavenge the DPPH radical was calculated from [(A_0_ – A_1_)/A_0_] × 100, where A_0_ is the absorbance of the control reaction (DPPH + Methanol) and A_1_ is the absorbance of the sample.

### 2.6 In silico experiments

#### 2.6.1 Protein preparation

For the current experiment, we have selected two enzymes of the cellular antioxidant mechanism, catalase and GR, respectively, for the demonstration of the inhibitory potential of the targeted chemical constituents of three mushrooms, that is, *G*. *lucidum*, *G*. *applanatum*, and *R*. *cajanderi*. Three‐dimensional (3D) structures of human erythrocyte catalase (PDB ID: 1DGH) and human GR (PDB ID: 1XAN) were downloaded from the Protein Data Bank (www.rcsb.org/pdb) in PDB format. The structures were prepared and refined using the Protein Preparation Wizard of Schrödinger‐Maestro v10.1. Charges and bond orders were assigned, hydrogens were added to the heavy atoms, selenomethionines were converted to methionines, and all waters were deleted. Using force field OPLS_2005, minimization was carried out, setting maximum heavy atom root‐mean‐square deviation to 0.30 Å.

#### 2.6.2 Ligand preparation

For molecular docking analysis, we have selected a total of 10 compounds from three respective mushrooms through the literature review. Of these, shushe acids A–D **(1–4)** were obtained from *G*. *applanatum*, **5** and **6** were selected from *R. cajanderi*, and ganoderiol *F* **(7)**, ganodermanondiol **(8)**, ganolucidic acid A **(9)**, and lucidumol B **(10)** were obtained from *G*. *lucidum* (Cör et al., 2018; He et al., 2006; Luo et al., 2017). Among the compounds, **1–6** were drawn using ChemDraw version 16.0 (PerkinElmer ChemOffice Professional), and **7–10** were downloaded from PubChem databasein.sdf format (CID: 471,008, 73,294, 475,412, and 475,411). In addition, we have used dihydro‐nicotinamide adenine dinucleotide phosphate (NADPH; compound CID: 5886) bound to PDB ID: 1DGH and flavin adenine dinucleotide (FAD; compound CID: 643,975) bound to PDB ID: 1XAN as positive control for this study. The 3D structures of the targeted compounds were constructed using LigPrep in Schrödinger Suite 2015 with the OPLS_2005 force field. The pH 7.0 ± 2.0 was used for the generation of ionization states of the compounds, which used Epik 2.2 in the Schrödinger suite. Up to 32 possible stereoisomers per ligand were retained.

#### 2.6.3 Receptor grid generation

Receptor grids were calculated for the prepared proteins for the observation of poses by various ligands, which bind within the active predicted site during the docking procedure. In Glide, grids were generated, keeping the default parameters of van der Waals scaling factor 1.00 and charge cutoff 0.25 subjected to the OPLS_2005 force field. A cubic box of specific dimensions centered on the centroid of the active site residues was obtained for the receptor. The bounding box was set to 14 × 14 × 14 Å for docking experiments.

#### 2.6.4 Glide standard precision ligand docking

Standard precision flexible ligand docking was carried out in Glide of Schrödinger‐Maestro v10.1 (Emon et al., 2020; Friesner et al., 2004, 2006) within which penalties were applied to non‐cis/trans amide bonds. For ligand atoms, van der Waals partial charge cutoff and scaling factor were selected to be 0.15 and 0.80, respectively. Final scoring was done on energy‐minimized poses and showed as Glide score. The best docked pose with the lowest Glide score was recorded for each ligand.

#### 2.6.5 Prime/molecular mechanics–generalized born surface area simulation

Prime/molecular mechanics–generalized born surface area (MM–GBSA) approach was used to calculate the binding energies of ligand along with ligand strain energies for a ligand and a single receptor. MM–GBSA is a method that combines OPLS–AA molecular mechanics energies (EMM), an SGB solvation model for polar solvation (GSGB), and a nonpolar solvation term (GNP) composed of the nonpolar solvent accessible surface area and van der Waals interactions (Adasme‐Carreño et al., 2014). Here, the Glide pose viewer file of the best conformation chosen was given as the source in prime/MM–GBSA simulation (Misini Ignjatović et al., 2016). The total free energy of binding is.

$$\Delta G_{\text{bind}}=G_{\text{complex}}‐\left(G_{\text{protein}}+G_{\text{ligand}}\right),$$

where G = EMM + GSGB + GNP.

### 2.7 Ligand‐based Absorption, Distribution, Metabolism, Elimination, Toxicity analysis

The pharmacokinetic properties of all the selected bioactive compounds were evaluated and screened for drug candidacy using Lipinski's rule of five (RO5) and Veber's rule (C. A. Lipinski et al., 2001; Veber et al., 2002). According to Lipinski's RO5, a compound may show optimal drug‐likeness if it fulfills at least four of the five criteria, namely, molecular weight (not more than 500 g/mol), hydrogen bond donors (≤5), hydrogen bond acceptors (≤10), lipophilicity (<5), and molar refractivity (between 40 and 130). The other filter we considered is Veber's rule, according to which the number of rotatable bonds (≤10) and topological polar surface area (TPSA; ≤140 Å²) in a compound must be within the specified limit to show drug‐like behavior. The web tool SwissADME, which is indicated as a very useful tool in drug discovery, was used to analyze the abovementioned properties of the selected compounds (Emon, Alam, Rudra, et al., 2021; Veber et al., 2002). Compounds passing both Lipinski's filter and Veber's filter can be considered as suitable candidates for new drug development.

### 2.8 Bioactivity prediction of the selected

The selected compounds were subjected to bioactivity calculations using an online validation tool, Molinspiration cheminformatics server (www.molinspiration.com; M. M. Alam, Rashid, et al., 2021; Emon, Alam, et al., 2020; Rakib et al., 2020). Molinspiration calculates the molecular property associated with the drug‐likeness and predicts the bioactivity including G protein‐coupled receptor (GPCR) ligand, ion channel inhibitor, nuclear receptor ligand, kinase inhibitor, protease inhibitor, and enzyme inhibitors. The calculated bioactivity score for each of the selected compounds was compared with the specific activity of each compound, and the results were compared with the standards. In case of organic molecules, the probability is if the bioactivity score is more than 0, then it is active; if it is between −5.0 and 0.0, then moderately active; and if the score is <−5.0, then it is inactive.

### 2.9 Statistical analysis

All experiments were carried out in triplicate. The data are presented as mean ± standard deviation. The significance of the percentage of scavenging effect of the extracts of mushrooms was determined by using the one‐way analysis of variance test, followed by Dunnett's *t*‐test (two sided) compared with the positive control. Values of *p* < .001 were considered significant. The data were analyzed using SPSS (Statistical Package for the Social Sciences) program (version 16.0, SPSS Inc., Chicago, IL, USA). The half‐maximal inhibitory concentration (IC_50_) values were calculated by nonlinear regression analysis [(log inhibitor) versus. response – Variable slope (four parameters)] with the use of GraphPad Prism software version 6.01 (GraphPad Software, San Diego, CA, USA), and the chart was also drawn using the same software.

## 3 RESULTS AND DISCUSSIONS

### 3.1 Phytochemical screening

Table 2 shows the phytochemical components present in the methanol extract of mushrooms. This phytochemical screening revealed the presence of flavonoids and steroids in the methanol extract of every mushroom. Alkaloids were absent in only *G*. *lucidum*, whereas glycosides were present in all except *L. squarrosulus*. This phytochemical analysis also showed that tannins were present in *L. squarrosulus*, *T. lactinea*, *D. confragosa*, and *G*. *lucidum*. The presence of carbohydrates was not detected only in *C*. *lactineus* and *D. confragosa*, among all other mushrooms. Saponins were present in all mushrooms, except *R*. *cajanderi* and *G*. *lucidum*. Mushrooms show different medicinal properties due to the presence of several phytoconstituents. Alkaloids are phytometabolites that contain various groups of nitrogen. Alkaloids exhibit strong pharmacological actions that include analgesic, anti‐inflammatory, antimalarial, antimicrobial, antiviral, anticancer, anti‐ageing, cerebro‐protective, muscle relaxant, sedatives, and stomatics effects (Bribi, 2018). Except for *C*. *lactineus* and *G*. *lucidum*, alkaloids were present in all mushrooms of this experiment. Glycosides are organic compounds formed of a sugar group (glycon) and a non‐sugar group (aglycon) linked together by a glycosidic bond. Glycosides are used as an analgesic, antirheumatic, antibiotic, cardiotonic, demulcent, and purgative agent (Kren & Martinkova, 2001). This study showed that glycosides were absent in only *L. squarrosulus*, among all mushrooms. The presence of steroids was determined in every mushroom. Steroids are widely used for the treatment of inflammation and several autoimmune diseases. Anesthesia can be induced by using steroids (Shaikh et al., 2012). Carbohydrate‐based therapeutics are widely used in the treatment of cardiovascular and hematological problems (Kilcoyne & Joshi, 2007). Carbohydrates were not detected only in *C*. *lactineus* and *D. confragosa*, among all other mushrooms. Saponins were present in *L. squarrosulus*, *D. concentrica*, *T. lactinea*, *D. confragosa*, and *G*. *applanatum* mushrooms. Saponins are one kind of mushroom glycosides, which possess various pharmacological properties such as antiviral, anti‐inflammatory, and anticarcinogenic activities (Lee et al., 2012).

**TABLE 2:** Phytochemical constituents present in the methanol extracts of the mushrooms

Secondary metabolite	MELS	MEDC^1^	METL	MEFC	MEDC^2^	MEGL	MEGA
Alkaloids	+	+	−	+	+	−	+
Glycosides	−	+	+	+	+	+	+
Steroids	+	+	+	+	+	+	+
Carbohydrates	+	+	−	+	−	+	+
Flavonoids	+	+	+	+	+	+	+
Tannins	+	−	+	−	+	+	−
Saponins	+	+	+	−	+	−	+

Abbreviations: −, negative result; +, positive result; MEDC^1^, methanolic extract of *Daldinia concentrica*; MEDC^2^, methanolic extract of *Daedaleopsis confragosa*; MEFC, methanolic extract of *Fomitopsis cajanderi*; MEGA, methanolic extract of *Ganoderma applanatum*; MEGL, methanolic extract of *Ganoderma lucidum*; MELS, methanolic extract of *Lentinus squarrosulus*; METL, methanolic extract of *Trametes lactinea*.

### 3.2 Antioxidant activity

**FIGURE 1:**
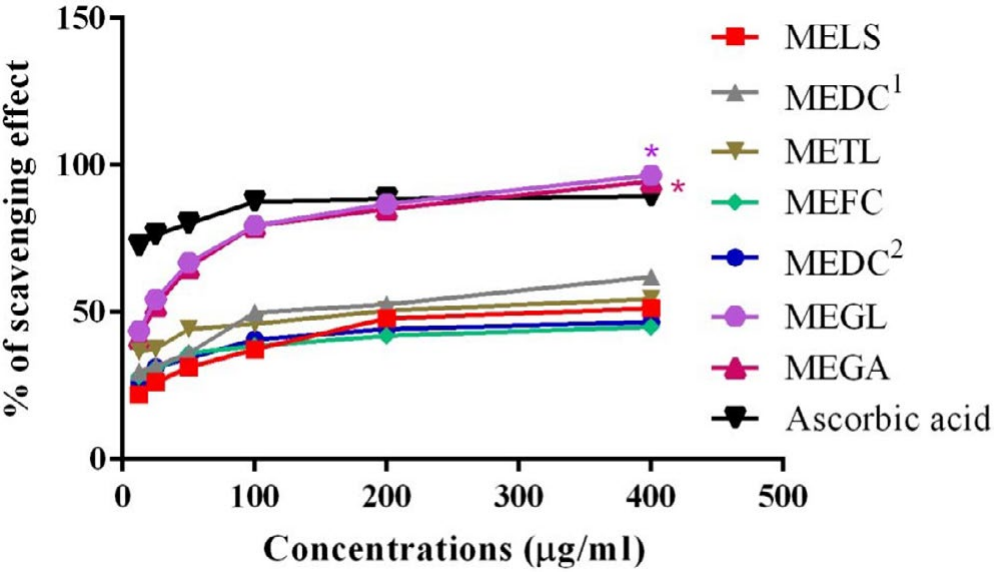
DPPH scavenging activity of wild mushroom species of the University of Chittagong campus. Abbreviations: MEDC^1^, methanolic extract of *Daldinia concentrica*; MEDC^2^, methanolic extract of *Daedaleopsis confragosa*; MEFC, methanolic extract of *Fomitopsis cajanderi*; MEGA, methanolic extract of *Ganoderma applanatum*; MEGL, methanolic extract of *Ganoderma lucidum*; MELS, methanolic extract of *Lentinus squarrosulus*; METL, methanolic extract of *Trametes lactinea*

At 400 μg/ml concentration, both *G*. *lucidum* and *G*. *applanatum* showed significant (*p* < .001) increase in the percentage of scavenging activity when compared with ascorbic acid. An increase in the scavenging activity of DPPH radical was found with the increasing concentration of the mushroom extracts (Figure 1). The results indicated that methanol extract of *G*. *lucidum*, *G*. *applanatum*, and *R*. *cajanderi* showed strong antioxidant activity with an IC_50_ values of 35.33, 38.73, and 39.44 μg/ml, respectively, in comparison with the IC_50_ value (49.19 μg/ml) of ascorbic acid. *D. confragosa* (51.21 μg/ml) showed almost similar antioxidant capacity as the ascorbic acid. The IC_50_ value of the remaining mushrooms was <100 μg/ml, except for *C*. *lactineus*. The IC_50_ values of all the mushroom extracts have been depicted in Table 3. Different pharmacological activities have shown phenolic compounds among which antioxidant and antimicrobial effects are more prominent (Bahri et al., 2014). Several reports have also suggested the utilization of flavonoids and many other phenolic compounds as free radical scavenging, anticancer, anti‐inflammatory, cardioprotective, and immune system promoting agents (Tungmunnithum et al., 2018). Flavonoids were present in every mushroom, whereas tannins were absent in *D. concentrica*, *R*. *cajanderi*, and *G*. *applanatum*. Due to the presence of phenolic compounds like flavonoids and tannins, every mushroom has shown free radical scavenging activity to some extent. DPPH is a free radical, which is stable and synthetic. A stable diamagnetic molecule is created when DPPH accepts an electron or hydrogen atom. The DPPH radical gives absorbance at 515–517 nm due to the presence of an odd electron in it. Because of this odd electron, it also produces a purple color solution in methanol. When this purple color solution is mixed with a substance with antioxidant molecules that can donate an electron or hydrogen atom, it becomes decolorized. As a result, there is a change has been observed in the absorbance. A lower absorbance at 517 nm indicates a higher radical scavenging activity of the extract (Boonsong et al., 2016; Hasan et al., 2009). A dose‐dependent increase in DPPH scavenging activity of all mushroom extracts is observed. A significant (*p* < .001) increase in the scavenging of DPPH radical is observed at 400 μg/ml concentration of both *G*. *lucidum* and *G*. *applanatum* with the comparison of positive control. Half‐maximal inhibitory concentration, IC_50_, is used to express antioxidant activity. It refers to the dose of antioxidant necessary to reduce by half of the initial DPPH radical concentration. So a lower IC_50_ represents higher antioxidant activity (Boligon et al., 2014; Kozarski et al., 2011). The results obtained from this study reported that a strong antioxidant activity with an IC_50_ values of 35.33, 38.73, and 39.44 μg/ml showed by *G*. *lucidum*, *G*. *applanatum*, and *R*. *cajanderi* mushrooms, respectively, when compared with the IC_50_ value (49.19 μg/ml) of positive control. *D. confragosa* showed an IC_50_ value of 51.21 μg/ml, which is almost similar to the IC_50_ value (49.19 μg/ml) of ascorbic acid. So *D. confragosa* has shown antioxidant capacity similar to ascorbic acid. The IC_50_ value of the remaining mushrooms was <100 μg/ml, except for *C*. *lactineus*, suggesting mild antioxidant activity compared with ascorbic acid. The major source of exogenous antioxidants is the phytochemicals of the mushrooms. Antioxidants can scavenge the reactive oxygen species, which are responsible for causing oxidative stress. At low concentrations, antioxidants can prevent the oxidation of substrate. Antioxidant is a great choice in the treatment of oxidative stress‐induced diseases such as cardiovascular diseases, cancer, and diabetes (Sarangarajan et al., 2017). Recent research has also shown that flavonoids and polyphenols are the most significant bioactive components of different kinds in medicinal mushrooms that have antioxidant capabilities against stress‐induced oxidative illnesses (Rehman & Akash, 2017). One of the main contributing factors in causing atherosclerosis is the oxidation of low‐density lipoprotein. Antioxidants block the oxidation of low‐density lipoprotein to prevent atherosclerosis (Shayganni et al., 2016). ROS are responsible for the promotion of cell migration and invasion in metastatic cancer cells. ROS scavenging potentials of antioxidants help to prevent the development of cancer (Fuchs‐Tarlovsky, 2013). Not only diabetes but also diabetic complications such as diabetic neuropathy are induced by oxidative stress. Superoxide, a ROS, contributes to the dysfunction of endothelial in diabetes mellitus. Antioxidants fight against oxidative stress in diabetes to reduce the hyperglycemic stage (Thakur et al., 2018). So the investigated mushrooms with antioxidants can be used to prevent diseases.

**TABLE 3:** Scavenging activity and IC_50_ values of mushroom extracts

Concentration	12.5 (μg/ml)	25 (μg/ml)	50 (μg/ml)	100 (μg/ml)	200 (μg/ml)	400 (μg/ml)	IC_50_ (μg/ml)
Samples	% Scavenging activity						
MELS	21.88 ± 0.28	26.23 ± 0.26	31.25 ± 0.42	37.36 ± 0.54	47.75 ± 0.80	51.16 ± 0.08	96.63
MEDC^1^	29.84 ± 0.49	31.69 ± 0.17	36.02 ± 0.24	49.77 ± 0.17	52.50 ± 0.14	61.97 ± 0.08	97.13
METL	36.62 ± 0.04	37.43 ± 0.21	44.21 ± 0.14	45.88 ± 0.11	50.44 ± 0.11	54.44 ± 0.12	112.40
MEFC	28.26 ± 0.14	30.88 ± 0.04	36.06 ± 0.04	38.40 ± 0.07	41.94 ± 0.14	44.93 ± 0.14	39.44
MEDC^2^	26.41 ± 0.29	31.30 ± 0.24	34.35 ± 0.04	40.63 ± 0.12	44.14 ± 0.08	46.57 ± 0.11	51.21
MEGL	43.50 ± 0.04	54.26 ± 0.04	66.83 ± 0.04	79.40 ± 0.04	86.85 ± 0.04	96.53 ± 0.07	35.33
MEGA	41.23 ± 0.04	52.20 ± 0.08	65.16 ± 0.04	79.35 ± 0.04	85.12 ± 0.04	94.47 ± 0.04	38.73
Ascorbic acid	72.41 ± 0.04	76.25 ± 0.00	80.02 ± 0.04	87.59 ± 0.04	88.38 ± 0.08	89.38 ± 0.00	49.19

Abbreviations: MEDC^1^, methanolic extract of *Daldinia concentrica*; MEDC^2^, methanolic extract of *Daedaleopsis confragosa*; MEFC, methanolic extract of *Fomitopsis cajanderi*; MEGA, methanolic extract of *Ganoderma applanatum*; MEGL, methanolic extract of *Ganoderma lucidum*; MELS, methanolic extract of *Lentinus squarrosulus*; METL, methanolic extract of *Trametes lactinea*.

### 3.3 In silico molecular docking analysis

**FIGURE 2:**
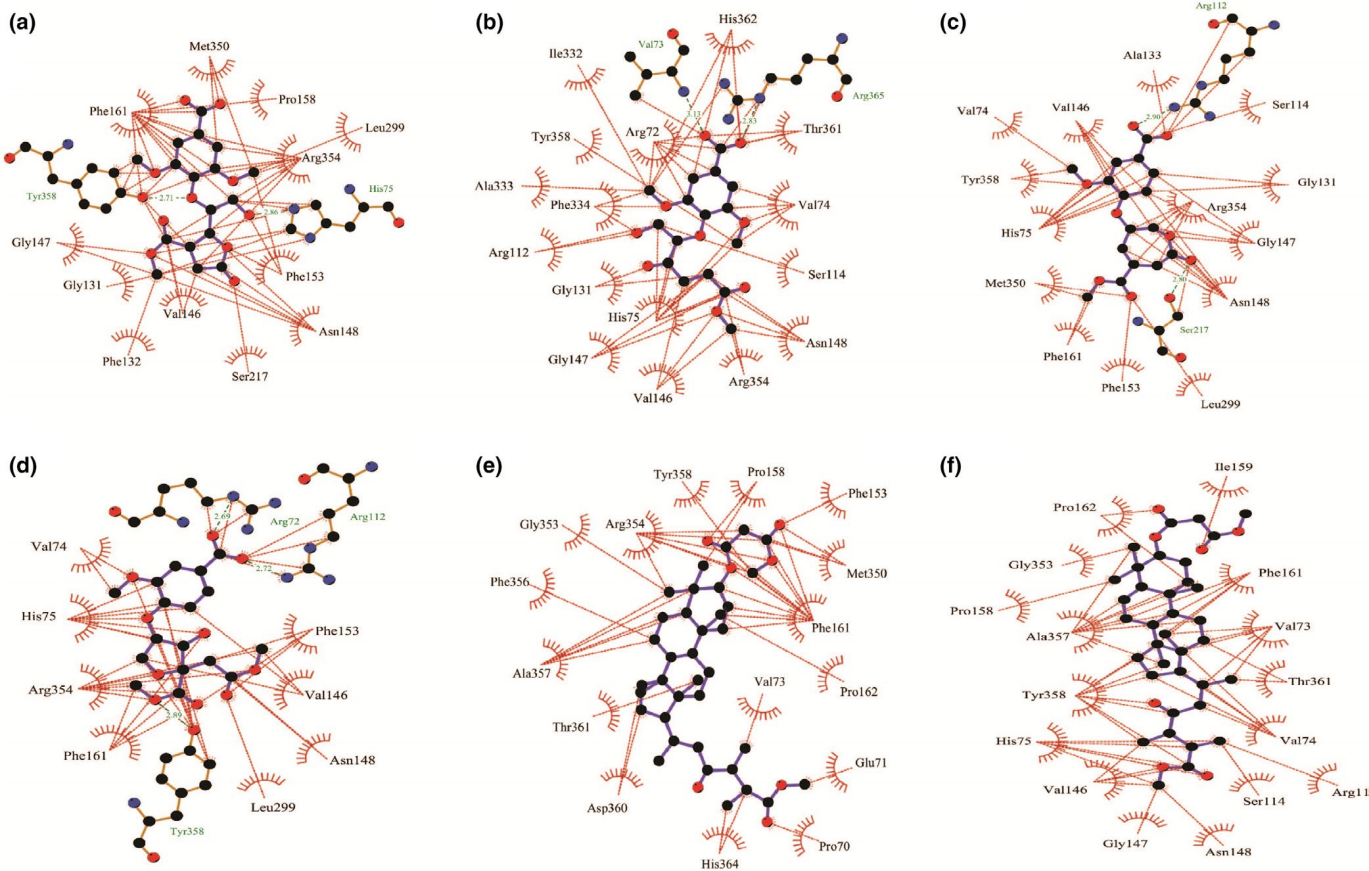
Representation of the interaction between (a) **1**, (b) **2**, (c) **3**, (d) **4**, (e) **5**, and (f) **6** with human erythrocyte catalase (PDB ID: 1DGH) through molecular docking simulation techniques. Green lines indicate interaction by hydrogen bonding, and red lines indicate the hydrophobic interaction. Figures were generated using LigPlot^+^ software

**FIGURE 3:**
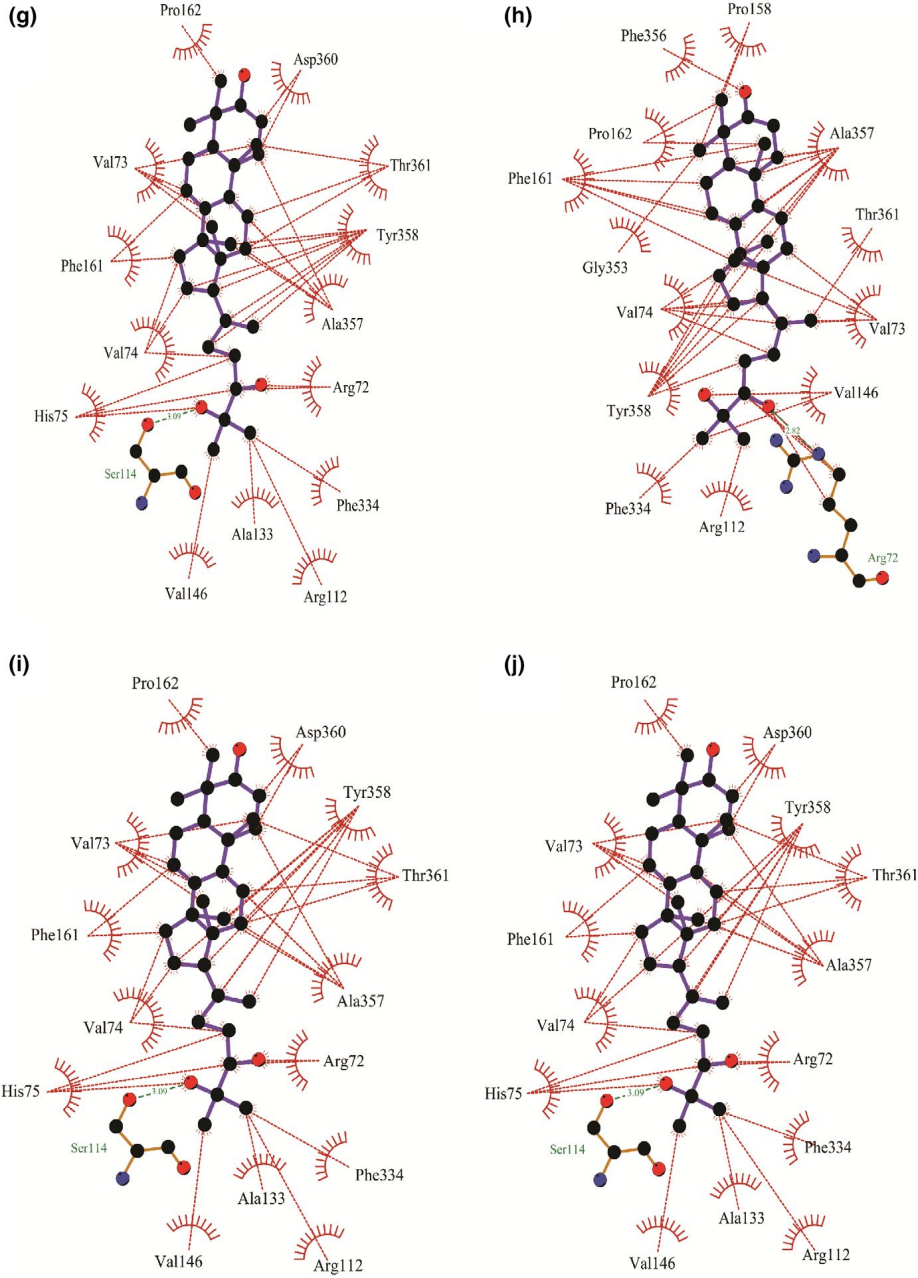
Representation of the interaction between (a) **7**, (b) **8**, (c) **9**, and (d) **10** with human erythrocyte catalase (PDB ID: 1DGH) through molecular docking simulation techniques. Green lines indicate interaction by hydrogen bonding, and red lines indicate the hydrophobic interaction. Figures were generated using LigPlot^+^ software

**FIGURE 4:**
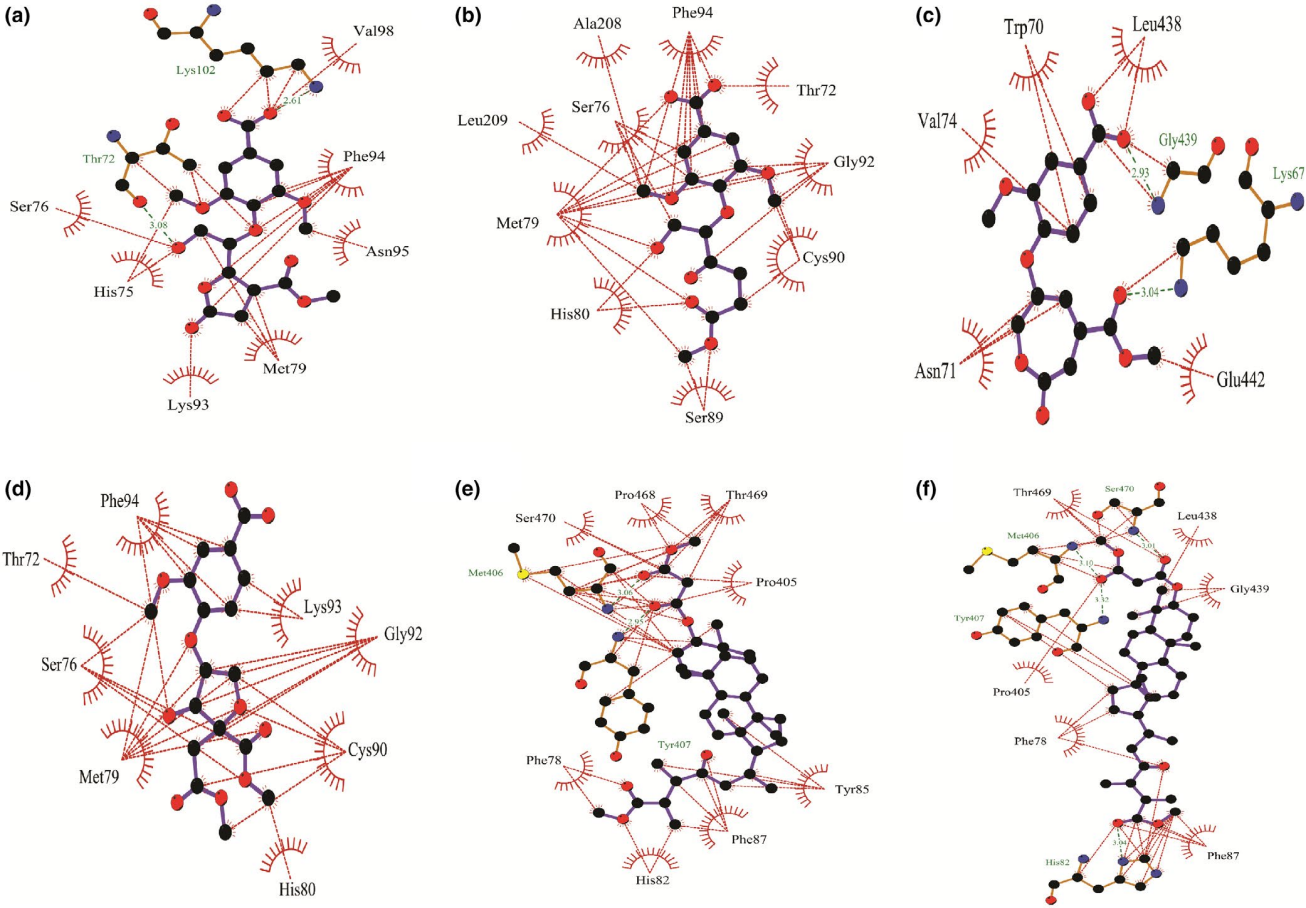
Representation of the interaction between (a) **1**, (b) **2**, (c) **3**, (d) **4**, (e) **5**, and (f) **6** with glutathione reductase (PDB ID: 1XAN) through molecular docking simulation techniques. Green lines indicate interaction by hydrogen bonding, and red lines indicate the hydrophobic interaction. Figures were generated using LigPlot^+^ software

**FIGURE 5:**
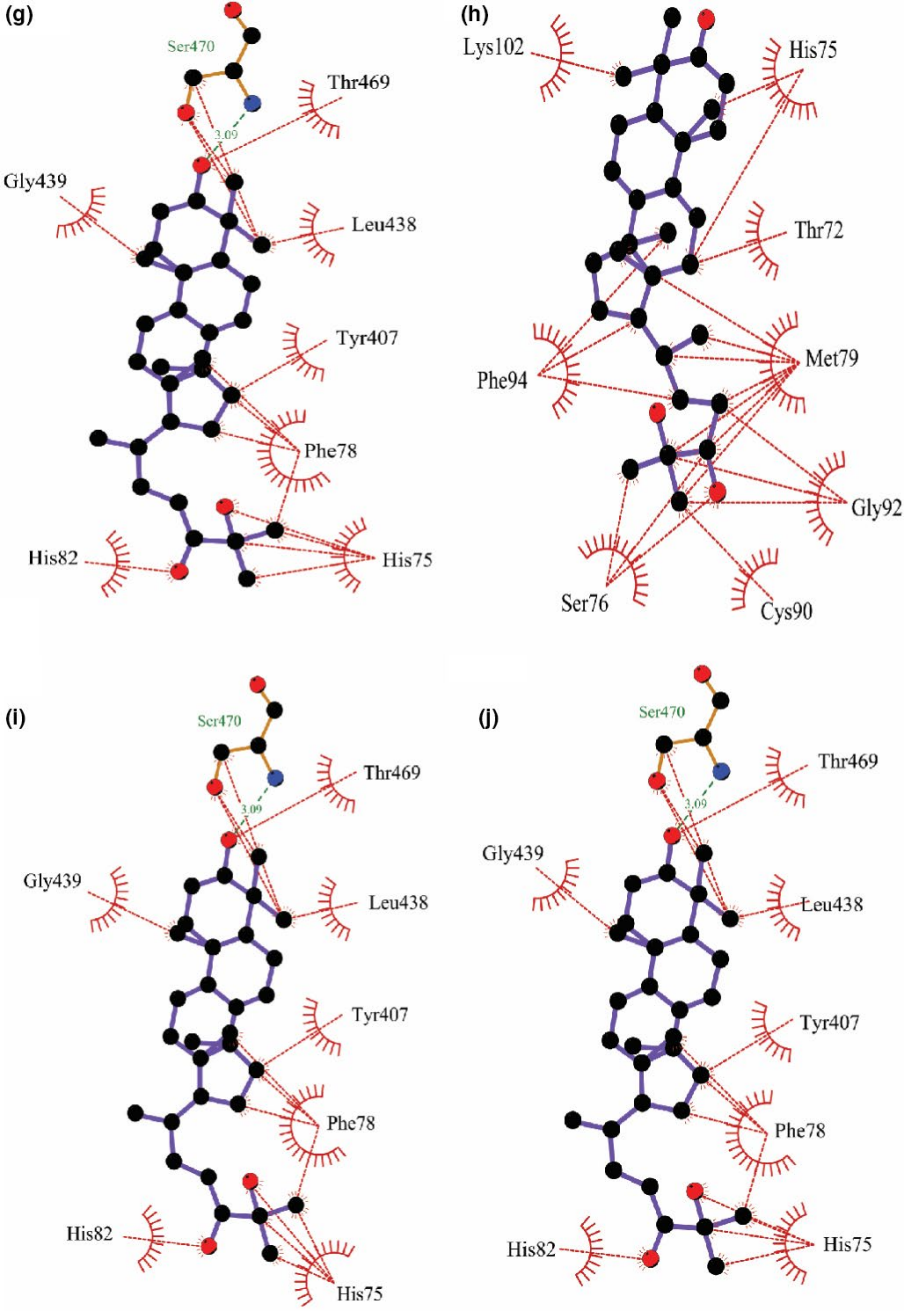
Representation of the interaction between (a) **7**, (b) **8**, (c) **9**, and (d) **10** with glutathione reductase (PDB ID: 1XAN) through molecular docking simulation techniques. Green lines indicate interaction by hydrogen bonding, and red lines indicate the hydrophobic interaction. Figures were generated using LigPlot^+^ software

**FIGURE 6:**
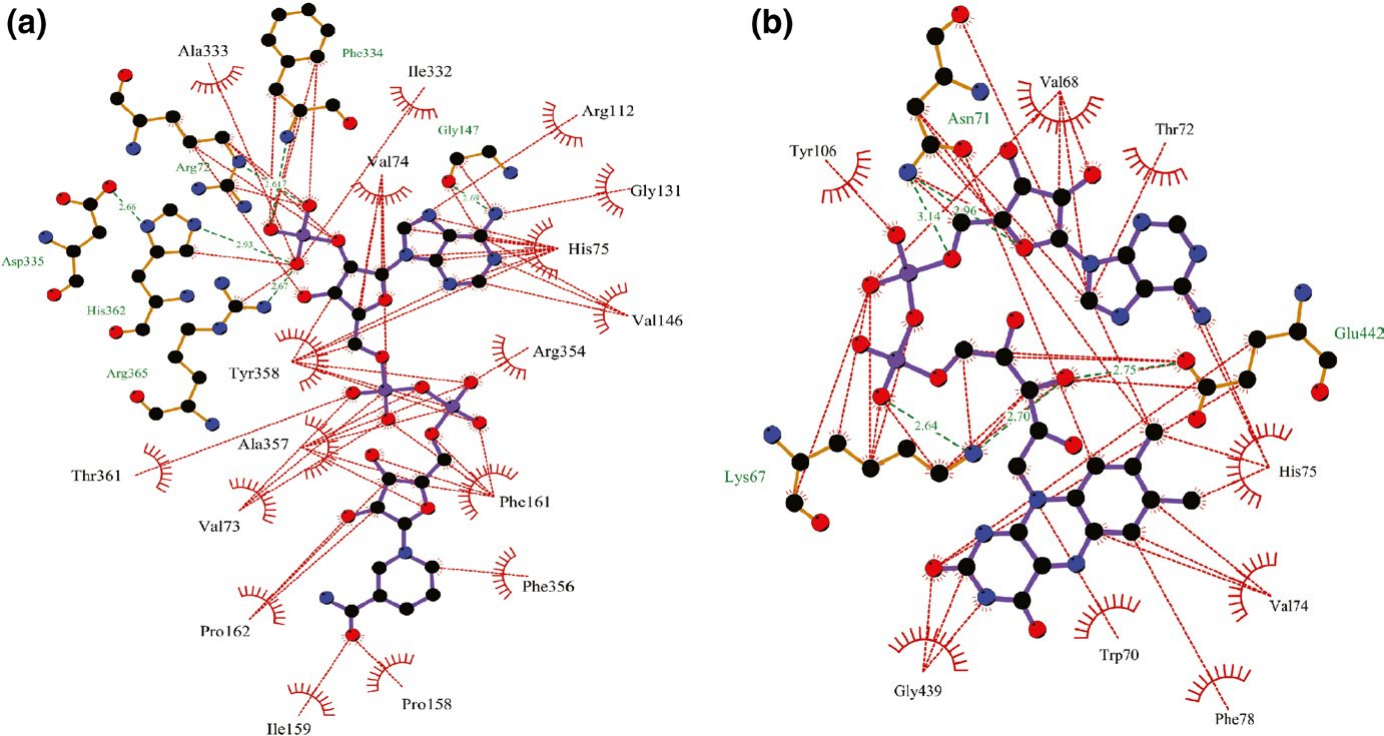
Representation of the interaction between (a) NADPH and (b) flavin adenine dinucleotide with 1DGH and 1XAN receptor, respectively, through molecular docking simulation techniques. Green lines indicate interaction by hydrogen bonding, and red lines indicate the hydrophobic interaction. Figures were generated using LigPlot^+^ software

Molecular docking analysis allowed us to identify the binding mode of the targeted ligand molecules with the selective receptor(s). The results of the molecular docking analysis were shown in Tables 4 and 5 and Figures 2, 3, 4, 5, 6.

**TABLE 4:** Molecular docking analysis between the selected compounds from *Ganoderma lucidum*, *Ganoderma applanatum*, and *Fomitopsis cajanderi* with human erythrocyte catalase (PDB ID: 1DGH)

PDB ID: 1DGH				
Compound	Binding affinity (kcal/mol)	ΔG_bind_ (kcal/mol)	Hydrogen bond residues	Hydrophobic bond residues
1	−5.7	−64.241	Tyr358, His75	Met350, Phe161, Pro158, Leu299, Arg354, Gly131, Gly147, Val146, Phe153, Phe132, Asn148, Ser217
2	−6.812	−56.533	Arg365, Val73	His362, Ile332, Thr361, Tyr358, Arg72, Ala333, Phe334, Val74, Arg112, Gly131, Ser114, His75, Gly147, Asn148, Arg354, Val146
3	−8.127	−59.734	Arg112, Ser217	Val74, Val146, Ala133, Ser114, Tyr358, Gly131, Gly147, Arg354, His75, Met350, Asn148, Phe153, Phe161, Leu299
4	−7.228	−58.275	Arg72, Arg112, Tyr358	Val74, His75, Phe153, Val146, Phe161, Arg354, Asn148, Leu299
5	−4.558	−70.374	—	His364, Asp360, Thr361, Ala357, Phe356, Gly353, Arg354, Tyr358, Pro158, Phe153, Met350, Phe161, Pro162, Val73, Glu71, Pro70
6	−7.921	−77.174	—	Pro162, Ile159, Pro158, Phe161, Gly353, Ala357, Val73, Tyr358, Thr361, His75, Val74, Val146, Ser114, Arg112, Gly147, Asn148
7	−4.851	−37.711	Ser114	Val74, Val73, Pro162, Asp360, Thr361, Phe161, Tyr 358, Ala357, Arg72, His75, Ala133, Phe334, Val146, Arg112
8	−6.799	−55.128	Arg72	Val74, Val73, Pro162, Pro158, Phe356, Phe161, Ala357, Gly353, Thr361, Tyr 358, Val146, Phe334, Arg112
9	−4.851	−37.711	Ser114	Val74, Val73, Pro162, Asp360, Thr361, Phe161, Tyr 358, Ala357, Arg72, His75, Ala133, Phe334, Val146, Arg112
10	−4.851	−37.711	Ser114	Val74, Val73, Pro162, Asp360, Thr361, Phe161, Tyr 358, Ala357, Arg72, His75, Ala133, Phe334, Val146, Arg112
*NADPH* (control)	−8.655		Asp335, His362, Arg365, Arg72, Phe334, Gly147	Ala333, Ile332, Arg112, Val74, Gly131, His75, Val146, Arg354, Phe161, Phe356, Pro158, Ile159, Pro162, Val73, Ala357, Thr361, Tyr358

**TABLE 5:** Molecular docking analysis between the selected compounds from *Ganoderma lucidum*, *Ganoderma applanatum*, and *Fomitopsis cajanderi* with human erythrocyte catalase (PDB ID: 1XAN)

PDB ID: 1XAN				
Compounds	Binding affinity (kcal/mol)	ΔG_bind_ (kcal/mol)	Hydrogen bond residues	Hydrophobic bond residues
1	−4.201	−55.937	Lys102, Thr72	Val98, Asn95, Phe94, Lys93, Met79, His75, Ser76
2	−4.222	−30.814	—	Leu209, Ala208, Phe94, Ser76, Thr72, Gly92, Cys90, Ser89, His80, Met79
3	−3.498	−24.114	Gly439, Lys67	Glu442, Leu438, Val74, Asn71, Trp70
4	−4.098	−91.384	—	Phe94, Lys93, Gly92, Cys90, His80, Met79, Ser76, Thr72
5	−5.307	−82.546	Met406, Tyr407	Ser470, Thr469, Pro468, Pro405, Tyr85, His82, Phe87, Phe78
6	−4.507	−77.449	Ser470, Tyr407, Met406, His82	Thr469, Leu438, Gly439, Pro405, Phe87, Phe78
7	−3.93	−66.634	Ser470	Thr469, Gly439, Leu438, Tyr407, His82, Phe78, His75
8	−4.327	−74.116	—	Lys102, Phe94, His75, Thr72, Met79, Ser76, Gly92, Cys90
9	−3.93	−66.634	Ser470	Thr469, Gly439, Leu438, Tyr407, Phe78, His75, His82
10	−3.93	−66.634	Ser470	Thr469, Gly439, Leu438, Tyr407, Phe78, His75, His82
*FAD (control)*	−7.27	—	Lys67, Asn71, Glu442	Tyr106, Val68, Thr72, His75, Val74, Trp70, Phe78, Gly439

Abbreviation: FAD, flavin adenine dinucleotide.

We have selected 10 ligands from the three respective mushrooms with the highest antioxidant activity and docked the targeted ligands with two respective enzymes of the human antioxidant system. In addition, we extracted the ligands bound with the published crystallographic structures of the two selected receptors, catalase and GR, respectively, and redocked with the respective protein to verify the docking procedure along with the scoring functions. The two control ligands, NADPH (bound with catalase; PDB ID: 1DGH) and FAD (bound with GR; PDB ID: 1XAN), interacted with the respective receptors and possessed the highest docking score. (The docking score was −8.655 kcal/mol for NADPH and −7.27 kcal/mol for FAD.) We observed that NADPH interacted with six amino acid residues (Asp335, His362, Arg365, Arg72, Phe334, and Gly147) through hydrogen bonding, and a total of 17 residues (Ala333, Ile332, Arg112, Val74, Gly131, His75, Val146, Arg354, Phe161, Phe356, Pro158, Ile159, Pro162, Val73, Ala357, Thr361, and Tyr358) were interacted by hydrophobic interaction while binding with catalase (Figure 6a). Compound **3** delineated the greater interaction towards the catalase enzyme, having a score of −8.127 kcal/mol (Table 4). However, **3** was only able to form two hydrogen bonds with residues Arg112 and Ser217, respectively, and surprisingly, like NADPH, **3** interacted with most of the residues (Val74, Val146, Ala133, Ser114, Tyr358, Gly131, Gly147, Arg354, His75, Met350, Asn148, Phe153, Phe161, and Leu299) through hydrophobic bonding (Figure 2c). Also, **6**, having the binding affinity of −7.921 kcal/mol, unlike NADPH, was unable to form any hydrogen bond with catalase but interacted with catalase through hydrophobic interaction with Pro162, Ile159, Pro158, Phe161, Gly353, Ala357, Val73, Tyr358, Thr361, His75, Val74, Val146, Ser114, Arg112, Gly147, and Asn148 residues (Figure 2f).

Further, FAD showed a binding affinity of −7.27 kcal/mol with GR and interacted with Lys67, Asn71, and Glu442 through hydrogen bonding, and Tyr106, Val68, Thr72, His75, Val74, Trp70, Phe78, and Gly439 residues were shown interacting through hydrophobic interaction (Figure 6b). The findings of the docking experiment with GR revealed that **5** showed the highest interaction with the enzyme (Table 5), possessing a docking score of −5.307 kcal/mol and interacted with Met406 and Tyr407 residues by hydrogen bonding, and Ser470, Thr469, Pro468, Pro405, Tyr85, His82, Phe87, and Phe78 residues represented hydrophobic interaction with GR (Figure 4e). Besides, despite showing a docking score of −4.507 kcal/mol, **6** was able to form four hydrogen bonds with the residues Ser470, Tyr407, Met406, and His82 (Figure 4f). Additionally, **6** interacted with six amino acid residues through hydrophobic attraction, and importantly, like FAD, it interacted with Phe78 residue.

To evaluate the clarification of the molecular docking simulation, we performed prime/MM–GBSA attributes to calculate the binding energy of the ligands with the enzymes. The results of the calculation of binding energies were shown in Tables 4 and 5. The results demonstrated that the binding energies of most of the ligands indicate a strong binding interaction towards the receptors. Biological research has been recognized worldwide as a promising field, and for this purpose, biomedical research requires a variety of informatics tools, including high‐throughput screening and database mining. A wide array of bioinformatics tools are recently introduced in the field of biological research, for instance, cancer metabolomics, epitope prediction, and secretome analysis. Lately, molecular docking is acknowledged as a key tool in molecular structural biology and the perspective of the molecular docking analysis includes the binding of the selected compounds (ligand molecules) with a selective protein (receptor; Morris & Lim‐Wilby, 2008). In the present study, we utilized molecular docking analysis for the identification of binding affinity among the selected compounds, which were isolated previously from *G*. *lucidum*, *G*. *applanatum*, and *R. cajanderi*, with catalase and GR, two crucial enzymes in the human antioxidant system. The docking experiment with the targeted compounds with catalase enzyme (PDB ID: 1DGH) showed that most of the compounds interacted with the amino acid residues like NADPH (control used for catalase), which has possessed the best docking score (−8.655 kcal/mol). Among the selected compounds, **3** and **6** were shown the highest docking score of −8.127 kcal/mol and −7.921 kcal/mol, respectively. The key interaction with NADPH and catalase enzyme includes hydrogen bonding with Asp335, His362, Arg365, Arg72, Phe334, and Gly147 residues and hydrophobic interactions with Ala333, Ile332, Arg112, Val74, Gly131, His75, Val146, Arg354, Phe161, Phe356, Pro158, Ile159, Pro162, Val73, Ala357, Thr361, and Tyr358 residues. Nonetheless, **3** yielded a hydrogen bond with Arg112 residue. A previous study already mentioned that Arg112 residue is responsible for salt bridge formation of the heme carboxylate radicals (Putnam et al., 2000). In addition, like NADPH, both **3** and **6** interacted with Tyr358 residue with hydrophobic interaction, and Tyr358 residue is responsible for the reactivity of electron donation towards the iron‐heme group of catalase (Putnam et al., 2000). Moreover, both compounds along with NADPH were interacted with Gly147 residue, which is an alignment with a previous study, showing that aminotriazole interacted with Gly147 residue (Sahoo et al., 2015). Importantly, Val74 and Phe153 are among the residues that are crucial for allowing small molecules to reach the heme molecule (Putnam et al., 2000). We found that **3** and **4** interacted with both the residues, whereas **2**, **6**, **7**, **9**, and NADPH interacted with only Val74 residue. Besides, **1–5** along with NADPH yielded hydrophobic interaction towards Arg354, and this amino acid residue might change the metal site by removing charges from Tyr358 residue. Additionally, Arg354 is not only responsible for reducing the charge repulsion, but also the multiple protonations of this amino acid residue are crucial for stabilizing oxidation produced electrostatic fields (Putnam et al., 2000). Further, we also have done molecular docking analysis for the targeted compounds with GR receptor (PDB ID: 1XAN), using FAD as the positive control, which possessed the best docking score of −7.27 kcal/mol. The key interaction includes three hydrogen bond interactions with Lys67, Asn71, and Glu442 residues, and hydrophobic interaction with Tyr106, Val68, Thr72, His75, Val74, Trp70, Phe78, and Gly439 residues. But **6** interacted with four hydrogen bonding with Ser470, Tyr407, Met406, and His82 residues, though the docking score of **6** was only −4.507 kcal/mol. Also, **5** yielded a hydrogen bond interaction with Met406 and Tyr407 residues. It has been reported that a hydrogen bond with Tyr407 residue is crucial for maintaining the human GR complex, and His82 is pivotal for the disulfide bond with Cys90. In addition, His82 along with His75, Phe78, Met79, and Asp81 contains the active site disulfide (Savvides & Karplus, 1996). Moreover, **1**, **7**, **8**, **9**, and **10** along with FAD interacted with His75 residue, **5**, **6**, **7**, **9**, and **10** as well as FAD interacted with Phe78 residue, **1**, **2**, **4**, and **8** interacted with Met79, and **5**, **6**, **7**, **9**, and **10** interacted with His82 residue.

For the verification of the docking experiments, we performed prime/MM–GBSA analysis, which is a groundbreaking quantum mechanics/molecular mechanics (QM/MM) attribute, to calculate the relative binding affinities utilizing best poses from a ligand–receptor interaction. We observed that most of the compounds yielded the highest binding score.

### 3.4 ADME/T analysis

We employed Lipinski's RO5 and Veber's rule to evaluate different absorption, distribution, metabolism and excretion (ADME) parameters of the selected biological compounds. According to Lipinski's RO5, **1**, **2**, **3**, **4**, **7**, and **8** met Lipinski's conditions as these compounds contravened no more than one rule. Conversely, **5**, **6**, **9**, and **10** failed to pass Lipinski's filter as it was found that the number of Lipinski's RO5 violations was two for each of these compounds. Here, **5**, **6**, and **9** violated rules of molecular weight and molar refractivity, whereas **10** violated lipophilicity and molar refractivity. In the case of Veber's rule, all the compounds except **2**, **5**, and **6** have passed Veber's filter as the number@ of rotatable bonds and TPSA was within the specified range for those compounds. So five compounds, namely, **1**, **3**, **4**, **7**, and **8**, have conformed to both Lipinski's RO5 and Veber's rules and may show optimal drug‐likeness in conformity with these rules. The results of the ADME/T analysis were shown in Table 6. In the current study, ADME analysis was done along with molecular docking simulation. The 10 selected compounds were further checked by online‐based prediction server SwissADME to explore their drug candidacy, pharmacokinetic parameters, and physicochemical properties. The pharmacokinetic properties of a drug candidate usually rely on the chemical descriptors of the molecules (Shahinozzaman et al., 2018). According to Lipinski’s rules of 5 (RO5), compounds violating any of the rules may have problems with permeability, absorption, and bioavailability as it was reported that compounds with lower molecular weight, hydrogen bond capacity, and lipophilicity may exhibit higher permeability (Duffy et al., 2015), better absorption, and bioavailability (Daina et al., 2014; Christopher A. Lipinski et al., 1997). Importantly, six compounds including **1**, **2**, **3**, **4**, **7**, and **8** exhibited orally active optimal drug‐likeness characteristics, according to Lipinski's RO5. In addition, the parameters involved in Veber's rule suggested that the number of rotatable bonds elicits molecular flexibility, which can be a good descriptor for potential drug candidates. On the contrary, passive molecular transport of drugs through membranes is expressed by the values of the TPSA (Shahinozzaman et al., 2018). Interestingly, almost all compounds except **2**, **5**, and **6** met the specified requirements under Veber's rule. After analyzing the results, we have found five compounds, namely **1**, **3**, **4**, **7**, and **8**, that satisfied the descriptors of both Lipinski's RO5 and Veber's rule, indicating that these compounds can be deliberated as potential drug molecules with receptor‐based optimization. The bioactivity score provides useful information on the binding cascade of a drug, which can describe the beneficial effects of the drug molecules inside the living body. A drug molecule is supposed to bind with a biological target, also referred to as drug target, and these drug targets are common proteins and can include enzymes, ion channels, and receptors. Molinspiration cheminformatics was utilized to predict bioactivity score for important drug targets like binding to GPCR ligand and nuclear receptor ligand, ion channel inhibition, kinase inhibition, protease inhibition, and enzyme activity inhibition. Importantly, the findings from the biological activity of our targeted compounds depicted no bio‐inactivity and moderate to high bioactivity towards all the parameters. Interestingly, the compounds passing both Lipinski's RO5 and Veber's filter were predicted to be compatible with the biological targets inside the living body in comparison with the controls.

**TABLE 6:** Drug‐likeness properties of the targeted compounds

Compound name	Lipinski's filter						Veber's filter	
	Molecular weight (g/mol)	Number of H‐bond acceptors	Number of H‐bond donors	MlogP	Molar refractivity	Rule of five violations	Number of rotatable bonds	TPSA (Å²)
	<500	≤10	≤5	<5	40–130	≤1	≤10	≤140
**1**	384.33	10	2	−0.23	88.14	0	9	137.82
**2**	356.32	9	2	−0.12	84.37	0	11	128.59
**3**	336.29	8	1	0.73	80.02	0	6	108.36
**4**	384.33	10	2	−0.50	87.58	0	9	137.82
**5**	600.83	7	0	4.99	169.25	2	12	95.97
**6**	599.82	7	0	4.99	168.19	2	12	95.97
**7**	454.68	3	2	4.82	137.93	1	6	57.53
**8**	456.70	3	2	4.91	138.44	1	5	57.53
**9**	500.67	6	2	3.06	139.89	2	6	108.74
**10**	458.72	5	3	5.01	139.40	2	5	60.69

Abbreviation: TPSA, topological polar surface area.

### 3.5 Bioactivity scores of the compounds

Molinspiration cheminformatics server was used to predict the bioactivity score for the selected compounds. NADP and FAD were taken as standard compounds. Selected compounds except **2**, **3**, **5**, and **6** were found to be highly bioactive towards GPCR ligands (>0), which indicates that they could bind more effectively with GPCR and were very close to the standard nicotinamide adenine dinucleotide phosphate (NADP) and better than FAD. The ion channel inhibiting property of **8** and **10** delineated more than the positive controls. Kinase inhibitor activities were observed to be moderate among the selected compounds, whereas binding to nuclear receptor ligand of all the compounds possessed greater bioactivity score in comparison with the controls. Besides, **1**, **4**, **8**, and **10** exhibited higher (>0) bioactivity values for protease inhibition and were close to the standard NADP and better than FAD. Interestingly, the enzyme inhibition score was high (>0) for all the compounds and close to the standard compounds as well. The findings of the biological activities were shown in Table 7.

**TABLE 7:** Biological activities prediction of the targeted compounds

Compounds	GPCR ligand	Ion channel inhibitor	Kinase inhibitor	Nuclear receptor ligand	Protease inhibitor	Enzyme inhibitor
Compound **1**	0.03	−0.14	−0.17	0.15	0.01	0.07
Compound **2**	−0.01	−0.26	−0.34	0.05	−0.03	0.11
Compound **3**	−0.13	−0.06	−0.43	0.12	−0.15	0.13
Compound **4**	0.11	−0.14	−0.12	0.23	0.14	0.30
Compound **FC4**	−0.17	−0.67	−0.84	0.18	−0.09	0.11
Compound **FC5**	−0.17	−0.67	−0.84	0.18	−0.09	0.11
Ganoderiol F	0.08	−0.16	−0.48	0.75	−0.01	0.53
Ganodermanondiol	0.11	0.02	−0.47	0.91	0.12	0.66
Ganolucidic acid A	0.03	−0.25	−0.80	0.76	−0.08	0.48
Lucidumol B	0.17	0.09	−0.29	0.92	0.16	0.69
NADP	0.53	−0.31	−0.18	−1.64	0.24	0.40
FAD	−0.36	−1.70	−1.17	−2.43	−0.57	−0.48

Abbreviations: FAD, flavin adenine dinucleotide; GPCR, G protein‐coupled receptor; NADP, Nicotinamide adenine dinucleotide phosphate.

## 4 CONCLUSION

The results of the present study exhibited that *G*. *lucidum*, *G*. *applanatum*, and *R. cajanderi* mushrooms exert strong antioxidant effects. This could be due to the presence of phenolic compounds like flavonoids and tannins in these mushrooms. Moreover, in silico studies also unleashed that the selective compounds from the mushrooms were biologically active and also interacted with the enzymes from the human antioxidant system. Furthermore, the selected compounds also possessed drug‐likeness properties. From the results stated in the preceding sections, we conclude that ROS and oxidative stress play a significant role in many consequences, whereas mushroom originated antioxidant therapy is one of the greatest ways to improve the adverse effects of oxidative stress. After that, further research on the phytoconstituents of the chosen mushrooms is recommended to understand the precise mechanism by which the mushrooms exhibit their antioxidant capabilities.

## AUTHOR CONTRIBUTIONS

**S. M. Moazzem Hossen:** conceptualization (equal); projectAdministration (equal); resources (equal); supervision (lead); validation (equal); writingReviewEditing (equal). **Mohammad Shahadat Hossain:** conceptualization (equal); dataCuration (equal); formalAnalysis (equal); investigation (equal); methodology (equal); software (equal); writingOriginalDraft (equal). **A. T. M. Yusuf:** dataCuration (supporting); formalAnalysis (supporting); investigation (supporting); methodology (supporting). **Priya Chaudhary:** dataCuration (supporting); investigation (supporting); methodology (supporting); validation (supporting); writingOriginalDraft (supporting). **Nazim Uddin Emon:** formalAnalysis (equal); investigation (equal); methodology (equal); software (equal); writingOriginalDraft (equal); writingReviewEditing (lead). **Pracheta Janmeda:** resources (equal); validation (equal); visualization (equal).

## CONFLICT OF INTEREST

The authors declare that they do not have any competing interests.

## ETHICAL APPROVAL

As this research works based on in vitro and in silico studies, ethical approval is not required.

## Data Availability

All the analyzed data are available in the manuscript.
